# Social Networks and Welfare in Future Animal Management

**DOI:** 10.3390/ani4010093

**Published:** 2014-03-17

**Authors:** Paul Koene, Bert Ipema

**Affiliations:** 1 Department of Animal Welfare, Wageningen UR Livestock Research, P.O. Box 65, 8200 AB Lelystad, The Netherlands; 2 Department of Farm Systems, Wageningen UR Livestock Research, P.O. Box 65, 8200 AB Lelystad, The Netherlands; E-Mail: bert.ipema@wur.nl

**Keywords:** Social Network Analysis, SNA, captive animals, animal management, approach-avoidance behavior, animal welfare, *Ursus arctos*, *Equus caballus*, *Gallus gallus domesticus*, *Bos taurus*

## Abstract

**Simple Summary:**

Living in a stable social environment is important to animals. Animal species have developed social behaviors and rules of approach and avoidance of conspecifics in order to co-exist. Animal species are kept or domesticated without explicit regard for their inherent social behavior and rules. Examples of social structures are provided for four species kept and managed by humans. This information is important for the welfare management of these species. In the near future, automatic measurement of social structures will provide a tool for daily welfare management together with nearest neighbor information.

**Abstract:**

It may become advantageous to keep human-managed animals in the social network groups to which they have adapted. Data concerning the social networks of farm animal species and their ancestors are scarce but essential to establishing the importance of a natural social network for farmed animal species. Social Network Analysis (SNA) facilitates the characterization of social networking at group, subgroup and individual levels. SNA is currently used for modeling the social behavior and management of wild animals and social welfare of zoo animals. It has been recognized for use with farm animals but has yet to be applied for management purposes. Currently, the main focus is on cattle, because in large groups (poultry), recording of individuals is expensive and the existence of social networks is uncertain due to on-farm restrictions. However, in many cases, a stable social network might be important to individual animal fitness, survival and welfare. For instance, when laying hens are not too densely housed, simple networks may be established. We describe here small social networks in horses, brown bears, laying hens and veal calves to illustrate the importance of measuring social networks among animals managed by humans. Emphasis is placed on the automatic measurement of identity, location, nearest neighbors and nearest neighbor distance for management purposes. It is concluded that social networks are important to the welfare of human-managed animal species and that welfare management based on automatic recordings will become available in the near future.

## 1. General Introduction

Hale [1] proposed that certain wild animal characteristics favored domestication. Indeed, domestic animals tend to be large, non-selective feeders that occupy open habitats. They are socially organized non-territorial species, typically occurring in relatively large groups in their natural environments. There are pros and cons to the grouping of animals. A strong social network is required to maintain the group and to allow them to cope with environmental challenges. In captivity, such networks may or may not exist, depending on actual group size and living conditions. In terms of evolution, it may be advantageous to keep animals in the groups to which they have become accustomed [2]. Data concerning social networks under farm animal species is scarce but is essential for the establishment of the importance of such networks. Additionally, deviations within a social network, such as an individual that gradually disassociates itself from the network, may provide essential information concerning health or welfare. 

Social Network Analysis (SNA) facilitates the characterization of network behavior and preferences at group, subgroup and individual levels. The study of animal social networks has become increasingly popular in many areas of behavioral research [3,4,5] and the temporal aspects of networks attract attention [6]; linking both individual behavior to population patterns and *vice versa* [7] to and personality [8]. Technical advances will make it possible to analyze whole populations in the near future [9,10,11]. Epidemiologists use SNA to model disease transfer and probably to understand the spreading of behavioral problems (*i.e.*, feather pecking in laying hens [12,13]). Application of SNA has improved our understanding of animal welfare in Atlantic salmons [14], rhesus macaques [15], pigtailed macaques [16], giraffes [17,18] and African elephants [19]. Knowledge concerning important or detrimental animals in the network forms a basis for management actions, such as removal of individuals from the group [5,16,20]. Removal of certain pigtailed macaques (*Macaca nemestrina*) resulted in decreased grooming, increased aggression and influenced the network structure [16].

Currently, social networks are often investigated for several reasons, one of which is the determination of animal social welfare [12,13,14,16,21]. Stable and suitable relationships support the social network and its individuals (social support [22,23,24]), while unstable and unsuitable networks may generate social stress [25,26,27]. In many cases, as with broilers or laying hens, groups are so large that nearest neighbors are probably strangers, casting doubt on the existence of a real social network and individual recognition. However, in many cases social networks might be important to animal fitness, survival and welfare [28,29]. Mismatches that have evolved within a species’ social network and the actual social network in a managed housing system should be observed and recorded [28]. Individual animals can react to correct mismatches, *i.e.*, approach or seek other animals or commodities (preferred) or flee to avoid other animals or commodities (non-preferred). 

Network analysis can reveal positive (individuals approach each other; social support; approaching) or negative (individuals avoid each other; social stress; avoiding) associations between individual animals (Mode-1 networks) or between individual animals and their environment (food, cover) or events (aggression, predation) in so-called “affiliation networks” (Mode-2 networks). Social networks describe and analyze the positive and negative associations between individuals. Conflicts between approach and avoidance tendencies in individuals may compromise animal welfare when not quickly and adequately resolved [30,31,32]. This may well be due to those animals displaying neither significant approach (towards neighbors) nor avoidance (of conspecifics) in the social network. 

We present some examples and data of small social networks in horses, brown bears, chicken and veal calves. In these four examples (#1–#4) social behavior, welfare and management are relevant. Simple management advice about removal (example#1—horses), management in large enclosures (example#2—brown bears), feather pecking (example#3—laying hens) and social housing (example#4—veal calves) are given. The four species are described to illustrate the social networks of social and solitary species in wild, semi-wild and domesticated conditions, under extensive or intensive management. The species were observed using various observation methods (nearest neighbor, x-y coordinates, visual, video and automated recording). Based on these differences, some hypotheses were formulated. Comparison of the examples may shed some light on differences in social networks of presumed solitary and social species, on space use and on the use of certain observation methods. Some general hypotheses have been formulated that will be elaborated further in the general discussion. The hypotheses are: (1) Solitary species have lower density networks than social species, (2) measurement of proximity (*i.e.*, nearest neighbors and/or nearest neighbor distances) is enough and supported with location measurements, (3) automated measurement of locations allows for quick measurement of nearest neighbor (NN), nearest neighbor distance (NND), locations, location of facilities and, consequently, changes in social networks can be determined on-line.

Nearest neighbor matrices can be made for each species, analyzed in a standard way using MatMan™ [33], UciNet [34] and NetDraw [35]. An example of nearest neighbor validation measurements using behavior observations in the horse is shown (Table 1). Further emphasis is placed on the importance of location for interpretation of nearest neighbor information in brown bears and laying hens and automatic measurement of location and/or nearest neighbors for management purposes using data on veal calves (Table 1). It is concluded that social networks are important to the welfare of captive animal species, and in the near future knowledge of social behavior and automatic recording will facilitate management.

**Table 1:** Overview of the presented Social Networks based on nearest neighbors of animals.

Example	#1	#2	#3	#4
Species	Equus caballus	Ursus arctos	Gallus gallus domesticus	Bos taurus
Example	Mares and foals	Dancing bears	Laying hens	Veal calves
Environment	Free range	Large bear enclosure	Stable	Stable
NN-measurement	Observer in the field	Observer in zoo	Video observations	Location sensors
Social life	Social	Solitary	Small groups, solitary	Social
SNA	1-mode	1-mode	1-mode, 2-mode	1-mode, 2-mode

## 2. General Methods—SNA

A social network consists of a number of nodes (individuals) and edges (relations between two nodes or individuals) [13]. Many concepts and parameters are developed to characterize and compare each node, detect relevant node subgroups within a network and characterize the network as a whole [36]. Basic input of Social Network Analysis (SNA) are associations between individuals; in this paper nearest neighbors associations or pairs are measured. 

Data of nearest neighbor pairs (X-Y coordinates or recording nearest neighbors) was collected using scan sampling. Data is selected from the daylight period in which all four species are the most active, although night activity in horses and bears is possible.

Associations between individuals were calculated initially using the simple ratio index [37] (frequency of being nearest neighbor divided by the total number of observations), but here the nearest neighbor (NN)-matrix was used, because all animals remained visible in every observation and thus the outcomes of both methods were the same. Preferred associations are nearest neighbor pairs that are observed (o) significantly more often than expected (e) according to the standardized residual (SR = (o-e)/√e), while pairs that avoid each other are observed less frequently than expected. The standardized residuals were calculated in MatMan™ [33]. These residuals showed significance at a P-value < 0.05 with standardized residuals of ≥1.96 and ≤ −1.96 [38]. Two matrices were constructed: (a) a positive matrix, in which positive significant associations (positive SR > 1.96) were determined with other values (SR < 1.96) set at zero and (b) a negative matrix in which negative significant associations (SR < −1.96) were determined (and made positive by multiplying SR by −1) with other values (SR > −1.96) set at zero. Both matrices provided weighted input for UciNet [34]. The resulting UciNet data file supplied input for UciNet or NetDraw [35] procedures. The resulting data can be interpreted in several ways (Table 2).

**Table 2:** Possible interpretations of nearest neighbor data from nearest neighbor matrices.

Standardized Residuals (SR)	>1.96	<−1.96
Social Network	Positive	Negative
Preference	Affection	Aversion
Motivation	Approach/seek	Avoidance/escape
Social behavior	Socio-positive (support?)	Socio-negative (stress?)
Individual welfare	Positive?	Negative?

The four animal species were monitored on a number of subsequent or consecutive days. Some parameters of SNA were calculated or visualized in NetDraw and UciNet: nodes (components of a network with known relationships, here individual animals), edges (also line or tie, is a relationship between two nodes of a network; strength is shown by line thickness) and resulting graphs (a set of nodes and edges, visualized as a picture showing dots connected by lines; labels in the graph are placed to the right of the nodes/individuals, thickness of the lines relative to SR-value). Arrows on the lines are directed, *i.e.*, the focal animal and/or nearest neighbor of the focal animal are indicated. Lines with two arrows indicate a bidirectional relationship (two-way); lines with a single arrow a unidirectional (one-way) relationship. A graph based on significant positive associations indicates a positive network; a graph based on significant negative associations indicates a negative network. Graphs are characterized by the group measure of density (calculated in UciNet; the proportion of all possible connections that are significant for the positive network (SR > 1.96) and for the negative network (SR < −1.96); the more significant the relationships, the higher the density.

The importance of the nodes (individuals) is often characterized by degree of centrality, betweenness centrality and closeness centrality [39,40]. Degree (number of connections a focal animal has with other group members), betweenness (the number of shortest paths between every pair of other group members on which the focal individual lies) and closeness (closeness is defined as the inverse of the farness; farness is defined as the sum of its distances to all other group members) are shown in the tables. In addition, subgroup information is given in the tables by cutpoints (which locate parts of the social network that would become disconnected if either an individual or connection were removed) and blocks (which are the resulting subgroups of individuals; the cutpoint is the individual that would, if removed, create the disconnection). Cutpoint and block presence (1) or absence (0) are given per individual. Cutpoints are biologically important individuals that should be removed to prevent disease transfer or that should not be removed to maintain the social network. Averages of node degree, betweenness, closeness, cutpoints and blocks are given in the tables; average cutpoint and block represent the proportion of individuals who are a cutpoint or part of a block. Data presented in this paper is output from NetDraw (within UciNet); from the *analysis* menu *centrality measures* and *blocks & cutpoints* have been activated; data from the *node attribute editor* was copied; NetDraw labels *farness* wrongly as *closeness*). Only undirected relationships are shown in the tables.

It is important for animals in a group to have stable and predictable relations with other animals in the group. The stability of a group is measured using the day-to-day variation of nearest neighbor relations in the group. The stability of the networks is thus given by the correlations between subsequent (and often consecutive) daily nearest neighbor matrices, calculated with the Mantel-test available in MatMan™ [33]. Significantly positive correlations imply network stability.

In addition to the social (1-mode) network, a 2-mode affiliation network (network exploring relationships between entities and events [12]) is given to show positive and negative significant individual-facility/commodity associations. In bipartite graphs for laying hens and veal calves examples are given of the positive or negative associations between individuals and facilities/commodities such as food, cubicles and nesting box. NetDraw provides graphs for these sub-structures and saves the information in the node attribute database. 

In the general discussion the networks determined for the four example species are compared using network densities. Additionally, the percentage of density in the positive network—compared with total density—provides a characterization of both the positive and negative networks. An indication of network stability is provided by the average of the day-to-day correlations of nearest neighbor matrices.

## 3. Example#1: Outdoor Horses—Direct Observation of Location

Much research has been performed on the social interactions and relationships between horses [41]. Age and order of arrival in the group are important determinants of rank. Within herds, horses often form stronger social affiliations with animals from similar social or age classes. Such peer attachments can last for several years during which the bond between mares appears stronger than between bachelor stallions. These strong long-term affiliations between mares contribute greatly to the stability of the herd. Affiliated animals participate in social activities such as mutual grooming and play and tolerate each other in close proximity [41,42]. Despite this knowledge on individual relationships, not much is known about the composition of the social network and existing bonds between horses. Other *equid* species have been the subject of SNA [43]. The lack of publications on SNA in the domestic horse (*Equus caballus*) is remarkable, despite the obvious potential for SNA [44]. We recorded nearest neighbors in horses in order to understand their social network to facilitate management of mare-foal relations. Some specific hypotheses were formulated. The lone dam (Mare2) has no relations with the foals. Foal1 is 3 yrs. of age and will be removed from the group; she is expected to have relations with the dams and few relations with the other foals.

### 3.1. Materials and Methods

Social networks of Dartmoor ponies were studied in 2011. The ponies, owned by Unifarm of Wageningen University, were kept at pasture at the Organic Experimental and Training Farm “Droevendaal”, the Netherlands. The herd under observation was kept outdoors under semi-feral conditions on an extensively managed pasture. The herd of mares comprised animals of different ages. The adult mares had been together in the Droevendaal herd since 2005. There were four dam/foal couples (mare and foal pairs that have the same numeric identifier) of which one was still nursing (Mare5-Foal5). Mare2 had no foal. The foals are of different ages Foal1 (3 yr.), Foal3 and Foal4 (2 yr.) and Foal5 (1 yr). An area of 4.35 ha extensively managed grassland was divided into 20 × 20 m plots (by numbered pavement tiles) to facilitate determination of the relative position of the ponies on the pasture. All animals were observed using continuous behavioral and simultaneous scan sampling. During continuous behavioral sampling, the social interactions “allogrooming” and “agonistic behavior” were recorded for a period of 50 hours. The location of the horse was recorded on paper with an image of the 20 × 20 m grid structure in the pasture. Coordinates (X-Y) were determined using scanned images of the mapped data and DataThief [45]. Nearest neighbors were determined using SpPack [46]. The method of analysis for the resulting NN-matrix is described in General methods—Paragraph 2. In order to determine a realistic image of the social network, the herd was observed for 8 days (*i.e.*, 440 scans per animal) during July 2011. In a subsequent study, the stability of the network was investigated further by daily removals of individual horses [47].

### 3.2. Results and Discussion

**Figure 1:**
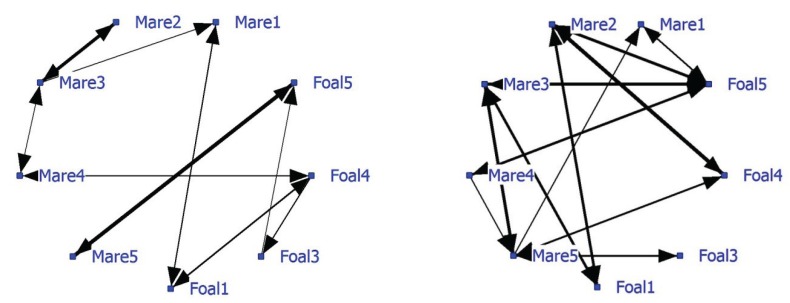
Social networks of Dartmoor ponies on extensive pasture, based on significant positive associations (left graph) and negative associations (right graph). Note that some individual attributes (mare/foal and their genetic relation) are given.

The average NND was 8.78 meter (SD of 8.98 meters). The network based on positive associations has a density of 0.21 and shows strong associations between Mare1-Foal1, Mare4-Foal4 and Mare5-Foal5 (Figure 1; left graph). Pair Mare3-Foal3 displays no significant relationship, but Mare2, Mare3 and Mare4 do. Foal4 has the most associations. The negative network, (Figure 1; right graph) density 0.31, shows that unrelated mares and foals avoid each other, while foals do not avoid each other and that Mare5 is often avoided. The strongest negative association is between Mare2 and Foal4 (thickest line).

Foal4 has the highest node degree, a higher betweenness and lower farness than the other foals (Table 3). Mare2 and Mare5 have few significant connections, low betweenness and a high farness; they are relatively isolated from the rest. Based on two cutpoints (Mare3 and Foal5) three subgroups are distinguished: Block1 (Mare2 and Mare3), Block2 (Mare5 and Foal5) and Block3, a rest group connected by Mare3 and Foal5.

**Table 3:** Social Network Analysis (SNA) characteristics for individual and subgroup characteristics of Dartmoor ponies in an extensive pasture.

ID	Degree	Betweenness	Farness	Cutpoint	Block 1	Block 2	Block 3
Foal1	2	4	27	0	0	0	1
Foal3	2	12	27	0	0	0	1
Foal4	3	16	24	0	0	0	1
Foal5	2	7	32	1	0	1	1
Mare1	2	2	29	0	0	0	1
Mare2	1	0	35	0	1	0	0
Mare3	3	8	28	1	1	0	1
Mare4	2	8	26	0	0	0	1
Mare5	1	0	39	0	0	1	0
Average	2.00	6.33	29.67	0.22	0.22	0.22	0.78
For cutpoint and blocks: 0 = no and 1 = yes							

The stability of the positive social network is shown by the positive significant correlations between NN-matrices on subsequent days and between 1 and 8 (Table 4).

**Table 4:** Nearest neighbor (NN)-Matrix correlations of eight consecutive days in Dartmoor ponies.

Days	1~2	2~3	3~4	4~5	5~6	6~7	7~8	1~8
Pearson’s r	0.71	0.48	0.27	0.41	0.55	0.65	0.74	0.71
P-value	***0.001***	***0.003***	***0.049***	***0.008***	***0.002***	***0.001***	***0.001***	***0.001***

Between-horse social interaction was also observed to provide a matrix of grooming and agonistic interactions. Social grooming and agonistic interactions were not related (R = −0.05, P = 0.644). The observed social grooming interactions correlated significantly with NN-matrix (R = 0.70, P = 0.000); agonistic interactions did not (R = 0.16, P = 0.077). The NN-matrix indicates that subjects that are close together show allogrooming and positive social behavior. When horses allogroom, a reduction in heart rate is observed which may indicate social support [48] and positive welfare.

### 3.3. Management

We were able to determine social networks based on nearest neighbor data for an all mare herd of nine horses. All animals had one or more preferred companions, and relationships were typically between kin or peers. The network consisted of three subgroups: a basic group of mares and foals, a pair of two adult mares and one mare-foal pair with the foal still sucking. Grooming frequency was found to be significantly positively correlated with this social network, which validates use of proximity measures to analyze social affiliation in this particular group. This relationship needs to be verified for different/other groups, populations and species. Aggressive interactions did not correlate significantly within the network. 

Foals were often sold at a young age. Foal1 (3 yr.) would be removed from the group, but still had significant positive relations with her mother and Foal4, and negative relations with Mare2 and Mare3. These findings are contrary to our expectations and hypothesis. On the other hand, Foal3 (2 yr.) no longer displayed a significant bond with her dam (Mare3) and showed only tentative dependencies with Foal4 and Foal5 and a socio-negative relationship with Mare5. Although Foal3 had a high betweenness, it was not a cutpoint and no separation issue was indicated, *i.e.*, removal had no influence on the subgroup structure. Based on these findings, it may be advisable to remove Foal3 instead of Foal1 from the group. The other foals maintained strong socio-positive bonds with their mothers, and removal of these foals presented a higher risk to the social structure and welfare of the group.

## 4. Example#2: Outdoor Brown Bears—Direct Observation of Location

Brown bears (*Ursus arctos*) are solitary animals except during breeding and cub rearing. In case of surplus food availability—salmon in rivers—large groups develop and a simple social organization develops [49,50]. Individuals typically have home ranges of 500–1500 square kilometers (males) and 100–800 square kilometers (females). Despite the fact that brown bears are territorial, their home ranges overlap, and boundaries are often not defended. In captivity brown bears are mostly kept in enclosures of a minimum of 400 m^2^ and bears seem to be non-territorial. Recently, large bear enclosures (LBE) have become popular, demanding more from zookeepers and management, *i.e.*, electric fencing, feeding, environmental and veterinary management. Some scientific research has been done in LBEs and is helpful in feeding and social management of bears [51,52,53]. The social interactions between brown bears have been studied [52] and may play an important role in the positive social network [53]. In large bear enclosures (LBE) many brown bears can be housed, and these supposedly solitary animals are forced to develop social contacts, especially in feeding situations. There is a lack of knowledge about the social network in such situations. One of the hypotheses investigated is that brown bears have no territories in LBEs.

### 4.1. Material & Methods

In 1995 detailed positions and behaviors were registered for 15 individual bears in a 2-ha bear forest at Rhenen in the Netherlands. These brown bears were of different ages and sexes (all neutered). They had been rescued from dancing shows, restaurants or inadequate zoos. Bear08, Bear09 and Bear10 came as former dancing bears from Turkey and were blind. The activities, locations and nearest neighbors (NN) of these brown bears were recorded by volunteers in a 10-day period under strict pre-programmed conditions using check sheets and maps on which data of behavior and location were recorded. Maps were digitized and x-y coordinates of locations recorded using DataThief [45] and nearest neighbors were determined using SpPack [46]. Subsequent analysis of the NN-matrix is described in Paragraph 2 (general methods).

### 4.2. Results and Discussion

**Figure 2:**
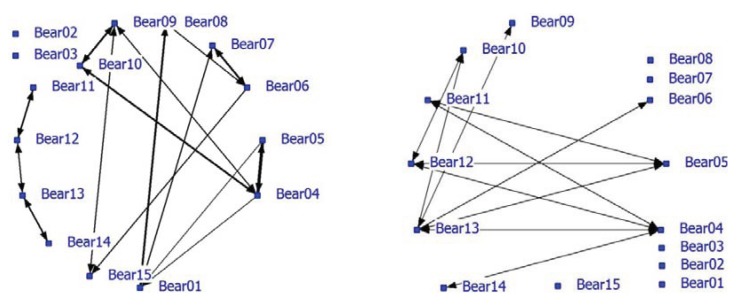
Social network of bears in a large bear enclosures (LBE) using a nearest neighbor distance (NND) smaller than 5 meter (positive network in left graph and negative network in right graph).

Significant positive associations were determined between some bears. The positive network showed a density of 0.16. The density of the negative network was 0.37 and was much higher than the density of the positive network. Nearest neighbor distances in the unlimited NN-matrix ranged from zero to 72 meter with an average of 13.36 meter (SD = 13.75 meters). Many individual bears appeared to have relatively fixed locations that may be territories or restricted home ranges and influence the associations and the network analysis. For instance, the blind Turkish bears (08, 09 and 10) occupied small areas that they hardly left. In a follow-up analysis, only nearest neighbors at a maximum NND of 5 meters were used (use of handmade notes on paper maps make a lower limit unreliable). Only 18% (638 of the 4710) of the NN-pair observations were used and the average NND was 3.38 meters (SD = 1.19 meter). The densities of the positive and negative network were 0.12 and 0.095, respectively. The correlation between the network using the NN-matrix with unlimited NND (N = 3491) and the network using max NND of 5 meters (N = 637) is 0.75 (P < 0.0001). The NND-limited network is shown in this paper (Figure 2). Bear02 and Bear03 were the only animals without any positive associations (solitary). Bears11, 12, 13 and 14 formed a pair-wise connected subgroup in the positive network (Figure 2; left graph; see also Table 5; Block 3, 4, 5) of young bears that roamed around with no fixed place in the enclosure. Furthermore, some strong pair associations (e.g., Bear04 and Bear05, Bear06 and Bear07, Bear01 and Bear09) were found. Bear09 and Bear10 were associated with Bear04. Negative associations were also found in the negative network (Figure 2; right graph). Six bears lacked negative associations while four bears had at least three negative associations (Bear04, Bear05, Bear12 and Bear13).

Bears 01 and 04 had the largest node degree and play a key role in the positive network (Table 5). Bears 02 and 03 have node degree zero, no betweenness and the highest farness score. The young bears (Bear11-14) are very distant from the other bears (Table 5: farness 169 and 171). Bears12 and 13 are cutpoints and important for the cohesion of the group of young bears. In nature, siblings often stay together after they have left their mother until they reach sexual maturity and become independent [54,55]. Bears 09 and 10 are associated; they knew each other before they came to the forest; both are blind and both like to play in the pond. Blind Bears 09 and 10 form a subgroup (Block 1) together with the very old male Bear01, Bears 04 and 05 (Block 2; betweenness high and farness low) and the females Bear06, Bear07 and Bear15. 

**Table 5:** SNA characteristics showing individual and subgroup characteristics of brown bears in the LBE.

ID	Degree	Betweenness	Farness	Cutpoint	Block 1	Block 2	Block 3	Block 4	Block 5
Bear01	4	9.17	103	0	1	1	0	0	0
Bear02	0	0	210	0	0	0	0	0	0
Bear03	0	0	210	0	0	0	0	0	0
Bear04	4	8.33	103	0	1	1	0	0	0
Bear05	2	0	106	0	1	1	0	0	0
Bear06	3	4.17	106	0	1	0	0	0	0
Bear07	2	1.67	106	0	1	0	0	0	0
Bear08	2	1.67	106	0	0	1	0	0	0
Bear09	3	4.67	105	0	1	0	0	0	0
Bear10	2	0	107	0	1	0	0	0	0
Bear11	1	0	171	0	0	0	0	0	1
Bear12	2	2	169	1	0	0	0	1	1
Bear13	2	2	169	1	0	0	1	1	0
Bear14	1	0	171	0	0	0	1	0	0
Bear15	2	3.33	106	0	1	0	0	0	0
Average	2.00	2.47	136.53	0.13	0.53	0.27	0.13	0.13	0.13
For cutpoint and blocks: 0 = no and 1 = yes									

The stability of the social network is shown by the positive significant correlations between the unlimited NN-matrices (N = 3491) on subsequent days and between relations on Days 1 and 10 (Table 6). Furthermore, the correlation between NN-matrices based on a maximum NND of 5 meters (N = 637) is also strongly positive (Table 6).

**Table 6:** Matrix correlations between NN-matrices based on daily nearest neighbor observations in brown bears for a period of 10 days. The last column shows the correlation between the first (1) and last day (10).

Days	1~2	2~3	3~4	4~5	5~6	6~7	7~8	8~9	9~10	1~10
Pearson’s r	0.77	0.79	0.78	0.82	0.75	0.70	0.83	0.73	0.75	0.69
P-value	*<0.001*	*<0.001*	*<0.001*	*<0.001*	*<0.001*	*<0.001*	*<0.001*	*<0.001*	*<0.001*	*<0.001*
NND ≤ 5m	0.78	0.77	0.53	0.84	0.74	0.62	0.85	0.70	0.69	0.75
P-value	*<0.001*	*<0.001*	*<0.001*	*<0.001*	*<0.001*	*<0.001*	*<0.001*	*<0.001*	*<0.001*	*<0.001*

Both series of positive significant correlations show the stability of NN-locations or possible territories of the brown bears and the stability of positive associations or preferences between the brown bears (Table 6). Despite the fact that bears are generally considered solitary, they might have preferred places (territories) and individuals as nearest neighbors. This is in agreement with a simple social organization in brown bears as described in high-density gatherings during salmon-fishing [49].

### 4.3. Management

Although brown bears are in most cases solitary animals, some strong positive associations between bears are observed in this forest group, although the network densities are low. The unlimited NND network might be based on the locations, home ranges or territories the bears occupy in the bear forest that have a more fixed position than expected. The networks based on NND of a maximum 5 meters indicate that the individuals have positive and negative associations or preferences. This implies that management of removals of bears or cleaning the area has to be aware of the social and positional relations; the social structure of the bear group has also to be taken into consideration for feeding (at different locations) and cleaning of the enclosure. In traditional zoo enclosures bears are probably unable to establish home ranges or territories. LBEs have the advantage of providing more space for the bears, but the opportunity to establish a fixed home range or territories can imply a disadvantage. An adult strategy of allowing solitary territoriality and a juvenile strategy of social clustering with mobility may provide the best options to avoid fights and injuries. The available space might still be too limited, restricting the opportunity to roam around, avoid or even escape other bears. Brown bear welfare may be ameliorated when the density of bears is low and enough space per bear is available.

## 5. Example#3: Indoor Laying Hens—Video Observation of Nearest Neighbor

The social structure of the ancestors (red jungle fowl) of laying hens (*Gallus gallus domesticus*) is not precisely clear, but hens live in small groups that may be territorial [56]. They have strong preferences for specific habitats and are seen mostly (80%) alone or in some cases in very small groups [57]. Based on their behavior in the wild, the social network of laying hens might be a group of individuals with few relations or even solitary individuals when housed together. Recently, it was investigated whether or not hens establish friendships [21] or associate for other reasons [58,59]. No consistent evidence was forthcoming from these studies of hens actively preferring others in their choice of companions or resource area. Laying hens cluster considerably in all kinds of environments [59]. Broiler chickens were more socially attracted than aversive/avoidant [60], but stocking density will play a large role in social associations [61]. The question remains whether or not hens grouped together function as individuals indifferent to each other or whether they have closer friendships within the group. As domestication changed aspects of the behavior of the chicken such as contra-freeloading [62], aspects of the social behavior might also be changed. Description of the social network of laying hens in captivity may display a social network, mismatches with networks seen in the wild and provide solutions for damaging behavior such as feather pecking [13]. Two specific hypotheses were investigated: (1) the social network of laying hens shows relatively many unconnected and solitary individuals, and (2) the overall social network of laying hens is correlated with feather pecking in the focal hens.

### 5.1. Materials and Methods

In this study recordings were used from a study on facility demand or use of commodities in laying hens [63]. For this research, eight Bovans Goldline commercial hens aged between 23 and 28 weeks were used. The hens were identified with a ‘backpack’ labeled with different symbols. The laying hens were housed in a pen, with the dimensions of 2.97 × 4.60 meters, at the experimental farm of Schothorst Feed Research BV (Lelystad, the Netherlands). The pen consisted of a central grid and litter area and contained eight nesting boxes (with a single perch in front of the entrances) at one end, three perches at the other end and two round feeders and one round drinker in the middle. Food and water were always available. Five continuous 24-hr video recordings of the hens were used from November 2006, recorded from above with an analogue camera situated in the middle of the pen. There were 16 hours of light in the pen per day. Scans were made every 10 minutes. This provided 96 scans per day and 480 scans per hen in total. Each scan included the nearest neighbor (NN), the nearest facility and the behavior of the focal hen. Nearest neighbor distances were not measured. Hens that were not visible were noted as Not Seen (NS). The facilities were the nest perch (NPerch), drinking area (Drink), feeding area (Feed), grid area (Grid), litter area (Litter), nests (Nest) and perches (Perch). A NN-matrix (1-mode SNA) and a hen-nearest facility matrix (2-mode SNA) were calculated. Subsequent analysis of the NN-matrix was as described in Paragraph 2 (General Methods).

### 5.2. Results and Discussion

**Figure 3:**
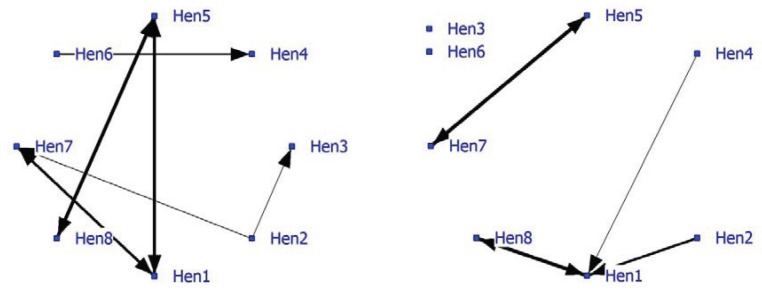
Social network of hens in an intensively managed indoor pen (positive network in left graph and negative network in right graph).

Relatively few significant nearest neighbors were found, so the density of the positive network was low at 0.16 (Figure 3; left graph). The graph shows some strong bonds especially between Hen5-Hen8, Hen1-Hen5 and Hen1-Hen7. The density of the negative network was lower (0.11). In the socio-negative graph, Hen1 displays the most connections often directed towards Hen8 (Figure 3; right graph). In socio-positive as well as in negative relationships, Hen1 may play a key role and may be important for the welfare in the group.

The importance of the individuals to the positive network is shown in individual and subgroup parameters (Table 7). Overall a low node degree was observed. Hen1 and Hen7 have the highest betweenness and the lowest farness within the network. Hen1 is also a cutpoint and connects Block3 (Hen1 and Hen7) with the hens in Block2 (Hen1 and Hen5). The other cutpoint is Hen5 who determines the relationship between Hen1 and Hen8. Subgroups based on cutpoints indicate three pairings of laying hens. Hens3, 4 and 6 are the most isolated hens in this group, having low degree, low betweenness, high farness and no subgroup participation.

**Table 7:** SNA characteristics showing individual and subgroup characteristics of laying hens in an indoor pen.

ID	Degree	Betweenness	Farness	Cutpoint	Block 1	Block 2	Block 3
Hen1	2	6	33	1	0	1	1
Hen2	2	4	35	0	0	0	0
Hen3	1	0	39	0	0	0	0
Hen4	1	0	57	0	0	0	0
Hen5	2	4	35	1	1	1	0
Hen6	1	0	57	0	0	0	0
Hen7	2	6	33	0	0	0	1
Hen8	1	0	39	0	1	0	0
Average	1.50	2.50	41.00	0.25	0.25	0.25	0.25
For cutpoint and blocks: 0 = no and 1 = yes							

The NN-matrixes of subsequent days are significantly positively correlated and display the stability of the social network of the laying hens (Table 8).

**Table 8:** Matrix correlations between NN-matrices of subsequent days in laying hens for 5 days. The last column shows the correlation between the first (1) and the last day (5).

Days	1~2	2~3	3~4	4~5	1~5
Pearson’s r	0.41	0.51	0.43	0.41	0.60
P-value	***0.009***	***0.002***	***0.005***	***0.014***	***0.000***

Laying hens show at least a number of stable individual associations, positive and negative. Whether they have friends [21] or not, or associate for other reasons [58,59] should be investigated further. The reason for this is that our study uses nearest neighbor analysis without a distance criterion and other studies have used the criterion that a hen is only a nearest neighbor when within a single bird-length of the focal hen [21]. Furthermore, in other research some specific periods of day and night were recorded while in the current study only daylight periods were recorded. The few significant associations found in captive conditions are in agreement with small groups of red jungle fowl associating under natural conditions [56,57]. Video recordings disclosed some limitations. The hens wore ‘backpacks’ with a symbol but often the hens flapped their wings and covered the symbol rendering them unrecognizable. With certain hens this happened quite often, affecting measurement reliability between hens. In future, this could be prevented by using an alternative recognition method. The hens were also less visible in the corners of the pen (this was probably due to the capability of the video or computer equipment). 

In addition to the nearest neighbor information, the behavior of the focal hen was recorded. The focal hens feather pecked 93 times when scanned. Hen5 (23), Hen4 (18), Hen2 (15) and Hen7 (14) showed most feather pecking, while Hen2 (40) and Hen1 (28) were most often the nearest neighbor and probably victims of feather pecking. The NN-matrix while the actor was feather pecking (N = 93) was significantly correlated (R = 0.30, P = 0.027) with the total NN-matrix (N = 2584), indicating that the overall NN-matrix might be influenced by the feather pecking relations. 

### 5.3. Management

**Figure 4:**
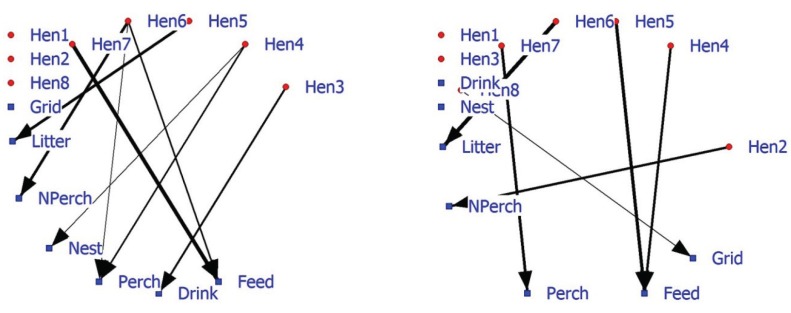
Affiliation (2-mode) network of eight hens and five commodities based on significant positive associations (left graph) or significant negative associations (right graph).

The social structure of laying hens based on nearest neighbor data may be relevant for understanding and reducing problem behavior in (large) flocks of laying hens. Behavior was scanned at sampling moments but not continuously (*i.e.*, feather pecking FP). The associations found in the social positive and negative networks are not in agreement with the preliminary data. Hen1 displays no FP and is more often than expected the NN in the positive network of Hen5 who shows most FP. In the near future, feather pecking interactions should be measured and related with positive and/or negative networks based on NN-matrices in laying hens. Previous research with these laying hens attempted to determine their facility requirements using discrete-event modeling and facility capacity under different housing environments [63]. SNA can also include facility associations alongside individual associations. The positive 2-mode hen-facility network depicts a number of hens (Hen1, Hen2, and Hen8) that show no preference (as nearest neighbor) towards facilities (Figure 4; left graph). Hens6 and 7 are more often in the vicinity of food. The 2-mode negative hen-facility network indicates that these hens are found less frequently in the vicinity of the perch (Hen7) and litter (Hen6) (Figure 4; right graph). Significant association between animals and facilities may improve our understanding of 1-mode networks and probably be combined in future research and management. 

In conclusion, 2-mode hen-facility networks show associations that are relevant for poultry farmers and managers. Information about (changes in) the associations of individuals with other individuals, facilities or events (f.i. own behavior or that of others) in the environment can be visualized and provide support to improve management of captive animals. A combination of 1-mode and 2-mode social networks provides a potential tool to estimate and judge social and facility demands of laying hens [64,65].

## 6. Example#4: Indoor Veal Calves—Automated Recording of Location

Under natural conditions, during the first two weeks of life, calves (*Bos taurus*) develop a strong life-long bond with their dam through suckling, grooming and vocalization. Occasionally, a dam may leave her calf in a “crèche” of peers [66]. On commercial farms cow and calf are usually separated within three days after birth. Modern on-farm dairy management is focused on productivity performance based on high stocking densities, early weaning and hand-rearing. The innate urge of the calf for social interaction is neglected. Current housing systems restrict normal behavior and communication with conspecifics and may compromise calf welfare [67,68,69]. Social requirements and capabilities are especially relevant to the interpretation of veal calf welfare whether single housed, small group-housed (five–seven individuals) or in larger groups (40–80 individuals) [70]. Recently, it is has been shown that calves appear to form preferential relationships before they are 3.5 months old. Therefore, housing cattle together from an early age could be beneficial [71]. For management purposes, the following hypotheses were investigated: (1) veal calves form a social network under intensive housing conditions based on preferences of nearest neighbors, *i.e.*, a positive social network, (2) group-housed calves of 3.5 month show stable associations, and (3) automated measurement of calf locations facilitates fast and precise social network analysis.

### 6.1. Material and Methods

Our study was performed with 10 Holstein-Friesian calves (six males/four females) aged 3 to 4 months (bodyweight 88–138 kg) at the Dairy Campus experimental farm (Leeuwarden, The Netherlands). The calves were housed in a pen consisting of a lying area (20 m^2^) with 12 cubicles, a walking area (25 m^2^) with a slatted floor and a feeding fence with 11 places. Individual location registration was performed per second from March 22 until April 17, 2013. This positioning determination system has been described previously [72]. Briefly, the system consists of receivers, transmitters and a processing computer, which together determine the position of each calf. The transmitters are placed at fixed locations throughout the barn, with a maximum distance of approximately 25 meters. The receivers are attached to the collar of the calves. The receiver determines the strength of the signal from each transmitter and sends this information to the processing unit. Signal loss is correlated with the distance between objects. These distances are used to calculate the position of the calves in the pen. Location is expressed as x- and y-coordinates in relation to the original location in the upper left corner of the barn where the pen is situated. Coordinate data became available per second and was corrected in a smoothing procedure [72]. Calf location was stored in the memory and adjusted when a subsequent position was recorded. Calculations were made of nearest neighbor and facility proximity every second. Data was sampled at a 10-minute interval to provide a NN-matrix based on observations of activity, feeding and lying [73]. Data were analyzed per day excluding the dark period (21–6 hrs.), when calf activity was low.

### 6.2. Results and Discussion

**Figure 5:**
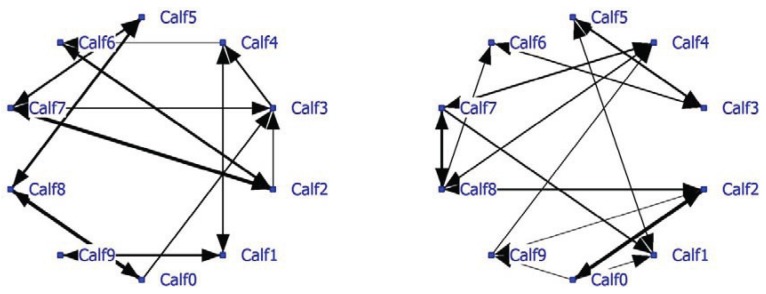
Social network of veal calves (NND < 1 meter) in an indoor intensively managed group (left: positive network, right: negative network).

A complete social network analysis was performed involving all data not limited by NND (see Example #2 brown bears). The positive network has a density of 0.22 and the negative network a density of 0.23. Data showed that often only one calf was eating and the others were lying down. In that case the nearest neighbor was one of the lying calves depending on the eating position. The social network was recalculated using only NN-pairs with a NND of less than 1 meter. The unlimited NN-matrix (N = 17,330) correlated significantly positively (R = 0.95, P < 0.0001) with the NN-matrix using NN-pairs of NND < 1 meter (N = 11,429). The sociogram of both analyses is essentially the same; the sociogram of positive calf associations at less than 1 meter is shown within the group of ten calves (Figure 5; left graph). All calves displayed positive relationships, mostly two-way and sometimes one-way (Calf0-Calf3 and Calf2-Calf3). The positive and negative network densities are 0.23. The same amount of significant negative associations was found between the calves (Figure 5; right graph).

Calf03 has the highest node degree and thus most connections with other calves and is important to the group social structure (Table 9). Its betweenness is the highest and its farness the lowest of the group. Calf03 is a member of subgroup Block1 including eight of the 10 calves. Calf01 and Calf04 are cutpoints and thus essential connectors for the three subgroups found, especially with regard to the somewhat isolated Calf09. If the cutpoints are removed, the social network falls apart and Calf09 becomes totally isolated.

**Table 9:** SNA characteristics showing individual and subgroup characteristics of veal calves in an indoor stable.

ID	Degree	Betweenness	Farness	Cutpoint	Block 1	Block 2	Block 3
Calf0	2	5.167	30	0	1	0	0
Calf1	2	8	33	1	0	1	1
Calf2	3	3.667	29	0	1	0	0
Calf3	4	17.667	25	0	1	0	0
Calf4	3	15.333	27	1	1	1	0
Calf5	2	1.833	33	0	1	0	0
Calf6	2	1.5	31	0	1	0	0
Calf7	3	6.833	28	0	1	0	0
Calf8	2	1	35	0	1	0	0
Calf9	1	0	41	0	0	0	1
Average	2.40	6.10	31.20	0.20	0.80	0.20	0.20
For cutpoint and blocks: 0 = no and 1 = yes							

Group stability is indicated by the correlations of NN-matrices between sample days (Table 10). Only Days 5 and 6 correlate significantly for the social network based on all data. The network structure appears to differ almost daily. Despite the indication of significant nearest neighbors, the overall analysis displays that the calves seem to have no preferred conspecifics. The observed network may be biased and based only on durations of cubicle lying behavior during long periods when the nearest neighbor is the same individual. In that case, calf location is relevant to the social structure. When the data are limited for only NND of 1 meter or less, the correlation between days remains generally not significant (Table 10). Only the correlation of the NN-matrices of Day 4 and Day 5 is significant, but negative. The almost complete lack of correlations between days places serious doubt on the existence of stable social relations within the group. 

**Table 10:** Matrix correlations between matrices based on daily nearest neighbor observations of veal calves for 12 days. The last column shows the correlation between the first and the last day.

Days	1~2	2~3	3~4	4~5	5~6	6~7	7~8	8~9	9~10	10~11	11~12	1~12
Pearson’s r	-0.09	0.00	0.15	-0.15	0.31	0.05	0.02	0.15	0.13	0.06	0.13	0.04
P-value	0.700	0.496	0.167	0.840	***0.024***	0.358	0.435	0.154	0.175	0.320	0.187	0.396
NND ≤ 1 m	-0.01	0.01	0.18	-0.32	0.20	0.07	0.04	0.14	0.09	0.07	0.15	−0.05
P-value	0.487	0.457	0.136	***0.011***	0.099	0.304	0.37	0.178	0.287	0.31	0.158	0.591

The claim that calves are capable of forming relationships within 3.5 months of age is challenged by the above findings [71]. Summarized over 12 days, the calves seem to have preferred neighbors, but the low non-significant day-to-day correlations show that these preferences are weak or non-existent.

### 6.3. Management

A positive and negative network was observed, but the day-to-day correlations were low and mostly not significant. This finding indicates that the stability of the social network is low or even that the networks may be based on chance caused by associations between calves using the same cubicles on consecutive days. In which case, location becomes very relevant for the interpretation of nearest neighbors in the same way as was found in the brown bear network, where fixed locations or territorial behavior were important factors. Especially for group-housed veal calves, natural management and consequent improvement of calf welfare, research in the day-to-day social network of the calves is crucial, and housing must be changed to facilitate social behavior and preferred associations between the calves.

**Figure 6:**
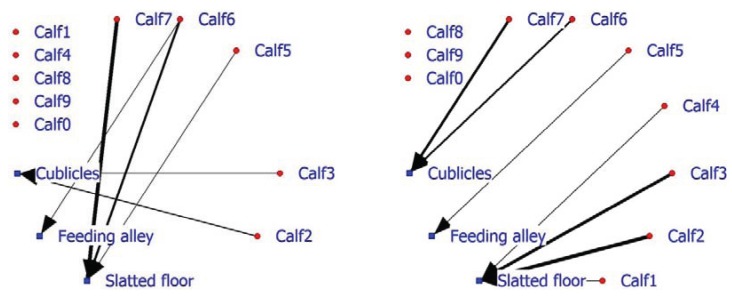
Social network of veal calves in an indoor intensively managed group (left: positive network, right: negative network).

In addition, facility usage is an important factor in on-farm management, as seen earlier in the laying hen example. The 2-mode SNA for veal calves indicates that in the positive network, half of the calves do not associate with a facility, while three calves display, above expectation, association with the slatted floor and two calves association with the cubicles (Figure 6; left graph). The negative calf-facility network indicates that three of the five calves (Calf8, Calf9 and Calf0) do not associate in a negative way with the facilities (Figure 6; right graph). Calf6 and Calf7 are found less frequently than expected in the cubicles and Calves1, 2 and 3 occupy the slatted floor less frequently than anticipated.

## 7. General Discussion

We used significant associations and dissociations based on standardized residuals of nearest neighbor frequencies as input for weighted positive or negative Social Network Analysis. This combination of old and modern methods is in our opinion suitable for the quick analysis of welfare-related problems. Replications of this method and comparison with different association measures or indices are needed to evaluate the method.

Information concerning nearest neighbors allows determination of significant associations between animals and between individuals and their environment. From our analysis, it is clear that this information remains insufficient. Validation is essential to the interpretation of the network, as is the location of each individual. We employed classic methods such as MatMan™ to find more individual and welfare related parameters. Most SNAs were performed with large datasets and based on un-weighted (1-0) edges between nodes. For small groups with small datasets, weighted SNA is probably more powerful. UciNet has some, albeit limited, power to analyze such weighted networks. Additional software for analyzing weighted social networks has only recently become available and is still under development [74,75]. Visualizing the observed network with NetDraw using weighted links or edges (SR) is helpful to judge the social network of small groups and can be used in management applications. The above examples show that SNA and knowledge concerning the social network of animals are relevant to the management and welfare of captive animals. 

Removal of animals from groups provides knowledge of the animals’ urges for social relationships and needs of the removed animal. The needs of the remaining animals must also be taken into consideration, for instance when an animal is removed, it may be crucial to the social structure or even create a cutpoint (breaking point) in the structure. Similar reasoning applies to introduction of animals into groups. We provide examples of networks based on nearest neighbor information. In horses, we found some indication that the positive network was indeed an important stimulant for individual welfare [48]. In the other examples no validation was observed. Fixed positions of animals in the enclosure or territorial behavior hamper the interpretation of the positive network in relation to welfare (e.g., bears and possibly the calf example). In order to reduce the influence of location preferences and fixed positions, the bear network was recalculated using a maximum NND of 5 meters and the calf network was recalculated using a maximum NND of 1 meter. These recalculated networks did not differ greatly from the complete networks using NN and unlimited NNDs. This also applies to negative networks and to the interpretation of negative effects on animal welfare. In calves, no day-to-day correlations between nearest neighbor matrices were observed, so consequently the resulting SNAs were difficult or impossible to interpret. It is possible that there is a social structure that is unstable or only stable for very short periods. Comparison between these findings and information from nature and literature is crucial to the welfare management of captive animals (see calves and crèches). At present, comparison of all the different housing and management conditions is impossible but may be possible in future, as more data from more species and more housing conditions become available. Automated measurement will be very helpful to facilitate applied Social Network Analysis. For the time being, the networks from the four examples have been compared using the densities of the positive and the negative networks provided (Table 11). 

The socio-positive networks show densities between 0.12 and 0.23 and might be indicative of social (horse 0.21 and calf 0.23) or solitary (bear 0.12 and hens 0.16) species. The negative networks also show higher densities in the social species (horse 0.31 and calf 0.23) than the solitary species (bear 0.10 and hens 0.11). These findings are in line with the formulated hypothesis. Probably, when enough space is available, animals use the space to avoid non-preferred individuals. The total densities of both the positive and negative networks show that in social species approximately half of the associations between individuals are clearly positive or negative and half is not explicit (Table 11). That is lower in laying hens and much lower in brown bears where three-quarters of the associations between animals do not differ from a random distribution. The parameter %Positive allows an investigation of the relative positive density. The most positive social network was identified in laying hens, followed by bears (NND < 5 m), calves and the horse network. Individual brown bears avoid each other more according to the unlimited bear network. As stated earlier this may be caused by the fixed locations, home range or territories of the bears. 

**Table 11:** Densities of the socio-positive and socio-negative networks of the four species, the total densities, the percentage of socio-positive of the total densities and the average correlation of the NN-matrices during trial periods.

Associations	Positive	Negative	Total	%Positive	Stability
Horse	0.21	0.31	0.51	40.53	0.54
Bear	0.16	0.37	0.53	30.63	0.77
Bear (NND < 5 m)	0.12	0.10	0.22	56.62	0.73
Chicken	0.16	0.11	0.27	60.01	0.44
Calf	0.22	0.23	0.46	48.78	0.07
Calf (NND < 1 m)	0.23	0.23	0.47	50.00	0.05

The stability of the SNs is shown in the average correlation between nearest neighbor matrices between days. Stability is highest in bears, followed by horses, laying hens and calves, where no stable social network was found.

However, there is room for improvement. Nearest neighbor identification is not always based on sufficient information for an adequate interpretation of the network data. Position, stability of the position and nearest neighbor distance is sometimes necessary for a correct interpretation of the SNs. In the first example, the nodes were labeled as mare or foal. In future, such node attributes may enhance the power of the SNA when information about, age, sex, weights or even personality is included as part of the SNA. This will become more likely with larger networks than the ones presented here. SNA provides opportunities to analyze the functioning of individuals in social groups in terms of approach and avoidance motivation and behavior. Using the SNA approach and avoidance tendencies in animals will provide additional information enabling determination of individual and group animal welfare.

## 8. Future Animal Management

Application of new technology, enabling simultaneous and more or less continuous localization of all individuals within a group, will make it possible to perform daily SNA. The parameters determined can then be translated into housing and management advice. In this context, parameters that reflect negative associations between individual animals or between individuals and their environment (facilities) become relevant, indicating that regrouping or removal of certain individuals can help animal welfare management. Social enrichment is one way of increasing welfare of solitary animals [76,77]. Social enrichment may strengthen a social network and increase individual welfare by introducing young animals [78]. Social enrichment by adding adults to social groups of juvenile animals may stabilize social networks of captives and increase welfare. In a situation where the social network is more or less stable from day to day, it can be determined how many and which animals have problems with the use of certain facilities such as places to rest, places to feed and other facilities within their environment. This information can lead to recommendations for adjusting the housing, available equipment and facilities. In addition, adjustments in the day-to-day management of the animals can be proposed, such as the feeding regime, or feed composition and quantity, and frequency of distribution. Current dairy farming practices already employ many different sensors to detect animals requiring special attention [79]. On-farm analyses of activity data are used to identify deviating activity, e.g., increased activity points to animals in estrus and reduced activity indicating health problems, such as locomotion. Daily information about the social network also allows following individual animals in time and thus providing an early warning of slow or sudden changes particularly in negative associations. It is interesting to consider whether or not the social network information can improve alerts for cows in estrus or with health problems. Application of location sensors in the management of farm animals is developing fast. These developments take place first in commercially important situations with large animals in small groups. In the near future, it is considered that it will become acceptable practice to apply sensors to pigs, and laying hens, if the analysis can be validated and the potential for animal welfare spin-off is made more apparent. Use of GPS or other location networks, will make SNA available for outdoor welfare management or even for feral animals. Certainly, intensive husbandry social network analysis has the potential to take on an important role in future animal management and welfare.
